# Integrating AI into Breast Reconstruction Surgery: Exploring Opportunities, Applications, and Challenges

**DOI:** 10.1177/22925503241292349

**Published:** 2024-11-06

**Authors:** Andrew Gorgy, Hong Hao Xu, Hassan El Hawary, Hillary Nepon, James Lee, Joshua Vorstenbosch

**Affiliations:** 1 Department of Plastic and Reconstructive Surgery, 54473 McGill University Health Center, Montreal, Quebec, Canada; 2 Faculty of Medicine, 4440 Laval University, Quebec City, Quebec, Canada

**Keywords:** artificial intelligence, innovation, plastic surgery, breast reconstruction, intelligence artificielle, innovation, chirurgie plastique, reconstruction mammaire

## Abstract

**Background:** Artificial intelligence (AI) has significantly influenced various sectors, including healthcare, by enhancing machine capabilities in assisting with human tasks. In surgical fields, where precision and timely decision-making are crucial, AI's integration could revolutionize clinical quality and health resource optimization. This study explores the current and future applications of AI technologies in reconstructive breast surgery, aiming for broader implementation. **Methods:** We conducted systematic reviews through PubMed, Web of Science, and Google Scholar using relevant keywords and MeSH terms. The focus was on the main AI subdisciplines: machine learning, computer vision, natural language processing, and robotics. This review includes studies discussing AI applications across preoperative, intraoperative, postoperative, and academic settings in breast plastic surgery. **Results:** AI is currently utilized preoperatively to predict surgical risks and outcomes, enhancing patient counseling and informed consent processes. During surgery, AI supports the identification of anatomical landmarks and dissection strategies and provides 3-dimensional visualizations. Robotic applications are promising for procedures like microsurgical anastomoses, flap harvesting, and dermal matrix anchoring. Postoperatively, AI predicts discharge times and customizes follow-up schedules, which improves resource allocation and patient management at home. Academically, AI offers personalized training feedback to surgical trainees and aids research in breast reconstruction. Despite these advancements, concerns regarding privacy, costs, and operational efficacy persist and are critically examined in this review. **Conclusions:** The application of AI in breast plastic and reconstructive surgery presents substantial benefits and diverse potentials. However, much remains to be explored and developed. This study aims to consolidate knowledge and encourage ongoing research and development within the field, thereby empowering the plastic surgery community to leverage AI technologies effectively and responsibly for advancing breast reconstruction surgery.

## Résumé

**Contexte:** L'intelligence artificielle (IA) a significativement influencé divers secteurs, y compris la santé, en améliorant les capacités des machines à assister dans les tâches humaines. Dans les domaines chirurgicaux, où la précision et la prise de décision rapide sont cruciales, l'intégration de l'IA pourrait révolutionner la qualité clinique et l'optimisation des ressources de santé. Cette étude explore les applications actuelles et futures des technologies de l'IA en chirurgie reconstructive mammaire, visant une mise en œuvre plus large. **Méthodes:** Nous avons mené des revues systématiques via PubMed, Web of Science et Google Scholar en utilisant des mots-clés et des termes MeSH pertinents. L'accent a été mis sur les principales sous-disciplines de l'IA : apprentissage automatique, vision par ordinateur, traitement du langage naturel et robotique. Cette revue inclut des études discutant des applications de l'IA dans les contextes préopératoire, peropératoire, postopératoire et académique en chirurgie plastique mammaire. **Résultats:** L'IA est actuellement utilisée en préopératoire pour prédire les risques et les résultats chirurgicaux, améliorant ainsi le conseil aux patients et les processus de consentement éclairé. Pendant la chirurgie, l'IA aide à l'identification des repères anatomiques, aux stratégies de dissection et fournit des visualisations tridimensionnelles. Les applications robotiques sont prometteuses pour des procédures telles que les anastomoses microchirurgicales, la récolte de lambeaux et l'ancrage de matrices dermiques. En postopératoire, l'IA prédit les temps de sortie et personnalise les calendriers de suivi, ce qui améliore l'allocation des ressources et la gestion des patients à domicile. Sur le plan académique, l'IA offre des retours personnalisés pour la formation des stagiaires chirurgicaux et aide à la recherche en reconstruction mammaire. Malgré ces avancées, les préoccupations concernant la vie privée, les coûts et l'efficacité opérationnelle persistent et sont examinées de manière critique dans cette revue. **Conclusions :** L'application de l'IA en chirurgie plastique et reconstructive mammaire présente des avantages substantiels et des potentiels divers. Cependant, beaucoup reste à explorer et à développer. Cette étude vise à consolider les connaissances et à encourager la recherche et le développement continus dans ce domaine, permettant ainsi à la communauté de chirurgie plastique d'utiliser efficacement et de manière responsable les technologies de l'IA pour faire avancer la chirurgie de reconstruction mammaire.

## Introduction

The modern world is at an apogee of technological advances. Machines are becoming more capable than ever through novel computing paradigms, with a noteworthy one being artificial intelligence (AI). AI may be best understood as the emulation of human intelligence and cognitive processes by computer systems, an idea incepted in the mid-20th century as Turing famously questioned: “Can machines think?”^1^ AI-powered machines leverage autonomous perception, synthesis, and inference of information in assisting with and accomplishing real-world tasks. Recently, the unveiling of ChatGPT (OpenAI), an AI-powered chatbot, represented yet another big step forward and only serves as a contemporary testament to the rapidly evolving nature of this field.

In healthcare, AI holds a prominent and promising role that has the potential to drastically improve care.^2^ Surgical specialties are unique in that they place great emphasis on hands-on manipulation, technical minutiae, and preoperative guidelines to assess risk factors and predict outcomes, making them potentially suitable candidates for incorporation of AI processes. Breast surgery is an especially diverse subfield, situated at the intersection of multiple surgical specialities who collaborate in a common, synergistic manner aiming to provide the best possible care for breast cancer patients. Trends in recent years depict increasing numbers of breast cancer diagnoses and breast reconstructions alike, making it an unequivocally key health service moving forward.^3,4^

Previous investigations have looked at AI in breast surgery but were mainly focused on breast cancer diagnosis and management, with little consideration to the reconstructive component.^5^ While the role of AI specifically to plastic surgery has been examined, breast reconstruction did not figure as a focal discussion point.^6^ In this context, it is the authors’ aim in the present study to scope the current literature in order to present a comprehensive review of applications of AI-based technologies on the plastic and reconstructive front of breast surgery. Modern examples, as well as perspectives on future directions, will be incorporated to streamline, bolster, and concretize theoretical and conceptual frameworks presented.

## Methods

A literature search of the PubMed database was performed using keywords and MeSH terms. AI-related terms included permutations of “artificial intelligence,” “machine learning,” “computer vision,” “natural language processing,” and “robotics.” These terms were searched in each of the following contexts: “preoperative,” “intraoperative,” “postoperative,” “education,” or “research.” Complementary searches using the same strategy were subsequently reproduced on Google Scholar and Web of Science for widened coverage. On top of systematic searches, cross-referencing allowed to uncover additional applications of AI, as well as applications in other fields that are of interest to breast reconstruction. Original and review articles pertaining to demonstrated applications of AI in breast reconstruction were considered; studies in other specialties were referenced in the discussion of future directions. We excluded opinion-based articles without original or demonstrated findings.

### AI Lexicology and Main Branches

It is worthwhile to lay out selected fundamental knowledge and clarify terminology to homogenize understanding of key points before delving into technical discussions. Machine learning, often mixed interchangeably with AI, is a subdiscipline that enables improvement of computer decision-making based on learning experience gained from vast amounts of data.^7,8^ Besides machine learning, AI also manifests within other frameworks. Through computer vision, computers can interact with the visual world by perceiving and processing inputs such as images and videos.^9^ Natural language processing (NLP), on the other hand, allows computers to understand human language in its written and/or spoken forms.^10^ Robotics provides computers with a tangible outlet to human reality, enabling them to manipulate physical objects by means of robots.^11^

**Figure 1.**
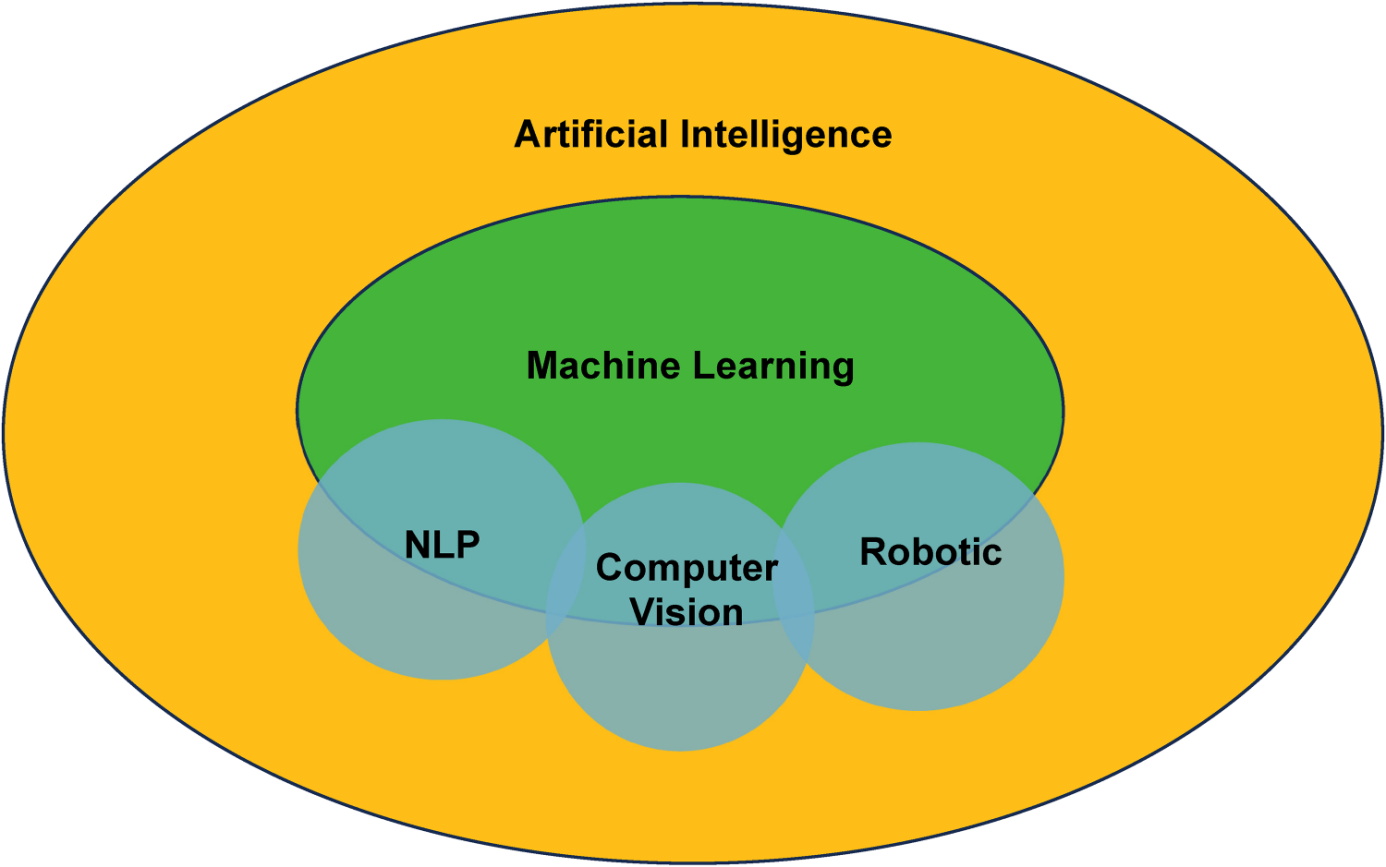
Artificial intelligence and its main subdisciplines.

These individual concepts should not be viewed as disconnected entities; in reality, they interact in complementary and synergistic fashions to achieve powerful results. Computer vision and NLP may be cross-integrated to enhance input perception capabilities of AI-powered agents and empower them to tackle increasingly complex tasks. Both technologies may be paired with robots for enhanced functionalities.^12^ The distribution of AI-related research is reflective of the high degree of interaction surrounding AI, as a preponderant body of studies are cross-disciplinary in nature.^13^ Figure 1 illustrates the dynamics between different branches of AI.

### Preoperative Applications

Breast reconstructive surgery begins well before the surgical act itself. Preoperative patient evaluation and counselling is essential for operative success, as diverging patient characteristics may dictate the plastic surgeon's choice of technique employed.^14^ To this end, O’Neill et al^15^ have developed a machine learning-powered prediction model to assess patients’ risk of experiencing free-flap failure following microsurgical anastomosis. Based on patient and procedural characteristics, the model was able to predict whether deep inferior epigastric perforator (DIEP) reconstruction would be complicated by flap failure. Myung et al^16^ have similarly utilized machine learning technology to predict donor site complications in free-flap breast reconstruction. Insights derived from these models may assist in the surgeon's choice of procedures by predicting postoperative morbidity given a patient's holistic profile.

Alternatively, even for a given surgical modality, machine learning prediction models can contribute to the informed consent process by providing patients with superior outcome forecasts than traditional methods.^17^ It is believed that machine learning is better situated than regression models to embrace the interconnectedness between individual risk factors for postoperative complications.^18^ A key distinction between the two is machine learning's ability to uncover trends and associations through autonomous learning, whereas regression models require prior subject knowledge for model specification.^19^ Indeed, Montemurro et al^20^ utilized machine learning to decipher risk factors for complications ensuing breast augmentation that were beforehand unknown. A recent study by Didzbalis et al^21^ demonstrated an alternative role of machine learning, whereby it analyzed online patient inquiries regarding mastopexy to better understand concerns surrounding the procedure. Likewise, Chartier et al^22^ designed an AI-based algorithm capable of simulating surgical results of breast augmentation using preoperative photographs. Ultimately, machine learning holds promise for improving preoperative patient counselling and thus expectation management, which alone is correlated with higher levels of satisfaction postoperatively.^23,24^

#### Future Directions

In breast reconstruction, preoperative models remain mostly tailored towards predicting outcomes based on patient data. With algorithmic refinement and sustained mass data collection, they could evolve into powerful tools paving way for precision medicine. From input of patient-specific information, algorithms can risk-stratify patients and propose tailored, individualized surgical management plans as well as testing/follow-up recommendations based on up-to-date scientific evidence and real-world data.^25^ In so doing, integrating other aspects of AI to machine learning, such as NLP, will allow algorithms to access vaster information, such as past health records and other databases, to perform big data analytics.^26^ By being integrated to major plastic surgery databases such as the Tracking Operations and Outcomes for Plastic Surgeons (TOPS) database, AI may help streamline data collection, organization, and analysis alike, overcoming current limitations of data registries and elevating evidence-based rigor in breast reconstructive care.^27^ The resultant is enhanced patient medical history consideration, risk mitigation, and individualized need identification,^28^ with modern AI paradigms demonstrating potential in uncovering social determinants of health within electronic medical records.^29^ Ultimately, based on a patient's holistic makeup and personal preferences, AI can be expected to comment on the optimal reconstructive modality (eg, autologous or alloplastic), fine-tune settings within a chosen modality (eg, flap/implant choice, incision type, anesthesia strategy, etc), as well as recommend custom follow-up plans according to personalized risk for complications.^30^

### Intraoperative Applications

The surgical act is akin to a well-choreographed dance, requiring careful planning before physically intervening on the patient. Even after the initial incision, every gesture remains intentional and calculated, rooted in pre-established protocols and clinical flair. However, anatomical variations and human imprecision present a challenge to the surgeon's ability to navigate the often-unpredictable operative environment. In response, AI may help elucidate surgical anatomy through storage and processing of large quantities of patient-specific data from preoperative imaging beyond human capacities. As an example, Mavioso et al^31^ introduced computer-assisted detection of vessel in free-flap autologous breast reconstruction, outperforming manual detection by the human eye. Likewise, machine-assisted operative agents, such as robots, may minimize, nay eliminate, intrinsic flaws of human operators during surgery, with the potential to better surgical results.

#### Robotic Surgery

Robotic surgery has also been gaining increasing traction. Great leaps have been made since the performance of stereotactic brain surgery by the PUMA 200 robot and laparoscopic cholecystectomy using the da Vinci system, both landmark events of the field.^32,33^ Noteworthily, the first robotic microsurgical anastomosis took place in the context of breast reconstruction.^34^ A 2017 systematic review by Dobbs et al^35^ identified microsurgery and flap harvest as the 2 aspects of breast surgery where robotic intervention was seen, though a significant proportion of related studies were constrained to the preclinical phase, suggesting nascency.^35^ A 2020 systematic review of robots in breast surgery revealed superposable findings while adding nipple-sparing mastectomy to the scope of application.^36^ More recently, a review by Chen et al^37^ identified a role for robotic assistance in implant-based reconstruction, where the da Vinci system was used to anchor the acellular dermal matrix to the chest wall.^38^ FDA approval of the first surgical robot dates back to 2000,^39^ and robot assistance has become the gold standard for selected procedures within certain specialties such as urology.^40^ In contrast to traditional human surgery, robotic surgery bears ergonomic benefits for surgeons, in addition to providing greater technical finesse by eliminating fundamental human flaws such as tremor and fatigue, leading to better postoperative outcomes.^41,42^ Nevertheless, there remains to date no FDA-approved robotic-assisted plastic surgery applications, in part attributable to the specialty's procedural variety and infrequent cavitary access.^43^ With significant foreshadowed expansion of the surgical robot market in coming years,^44^ maintenance of the status quo will likely be short-lived. Joint efforts with the FDA have already been undertaken for approval of robotic-assisted flap harvest for breast reconstruction, showing favourable outcomes.^43^ Along with preliminary data supporting long-term safety of robotic oncologic surgery, consensus from expert panels is starting to take form.^45,46^ Innovative microsurgical robots are also being pushed out, namely, MicroSure's MUSA (Eindhoven, Netherlands) and the MMI's Symani surgical system (Calci, Italy), both holding preliminary clinical promise for supermicrosurgical manipulations such as lymphatic anastomoses and with green light for clinical application in Europe.^47,48^ The additional precision conferred by robots becomes exponentially impactful as operative structures diminish in size, making them a natural fit for the microsurgical niche. Surgical robots can be expected to be made *intelligent*, as they grow equipped with the capacity to *see* (computer vision) and *converse* with the human surgeon (natural language processing), all while constantly self-adapting and self-improving from feedback (machine learning). Nevertheless, lingering cost barriers must be overcame,^49^ and further multicentric evaluations remain necessary.^47,48^

#### Intraoperative Guidance

AI's role in the intraoperative arena spans beyond robotic assistance. Novel research has been able to marry machine learning algorithms and ultrasound-based navigation to segmentate healthy and malignant tissue in real time, enhancing tumor resection.^50,51^ Augmented reality (AR), though technically not encompassed by the AI umbrella, is a complementary technology whereby digital information is superimposed onto one's vision of the real world.^52^ It was first brought into the operating room in the context of orthopaedic, craniofacial, and spine surgeries.^53–56^ AR headsets are slowly making their entry for breast surgery, where they have been employed for intraoperative tumor localization^57^ and 3-dimensional visualization.^58,59^ Interviews with plastic surgeons highlighted real-time anatomical visualization as the focal interest point for AR.^59^ With a myriad of AR tools being currently developed—Holosurgical's ARAI, the VOSTARS system, Augmedics, Microsoft HoloLens, Google Glass, Magic Leap goggles, Apple's newly released Vision Pro, among others—more applications are expected to arise moving forwards. Recently, HoloLens’ role in breast reconstruction has been demonstrated—it superimposed computed tomography angiography imaging onto patients’ physical bodies, facilitating identification and dissection of perforators.^60^

#### Future Directions

Intraoperatively, robotic breast surgery has huge untapped potential. A wider diversity of robotic machinery is being designed and tested, such as robotic microscopes, with or without integrated AR capabilities.^61^ The RoboticScope by BHS, approved for clinical use in Europe, has applications in both lymphatic and vascular anastomoses.^62,63^ There has also been notions of robotic scrub nurses which can potentially optimize operating room workflow.^64^ It is also prudent to suggest a future potential to equip surgical robots with computer vision or autonomous learning technologies, which would unlock higher levels of intelligence and autonomy, making robots able to perceive and adapt in real-time to the dynamic surgical environment.^65^ To this end, there is a definitive need for unified data efforts to make available robust and standardized surgical data to facilitate and streamline training of intelligent surgical machinery.^66^ With compelling success stories in other surgical specialties,^67^ advancement of robotics and AR in breast surgery is possible, requiring interdisciplinary and cross-disciplinary collaboration between surgeons, radiologists, and scientists/engineers to push bio-entrepreneurial frontiers.^68,69^ Though autonomous robotic surgery has been reported,^70^ it is prudent to suggest that current focus be concentrated on consolidating evidence on robotic assistance before delving into robotic autonomy, which entails additional ethical considerations.

### Postoperative Applications

Though specific postoperative applications of AI in breast reconstruction were absent from the literature, AI-powered chatbots have recently been investigated in the context of diagnosing, triaging, and managing complications following aesthetic breast plastic surgery.^71^ In orthopaedics, AI was used to develop an agent capable of selecting personalized follow-up times based on individual needs and vocally communicating them to patients in a humanly manner.^72^ Similar agents have been designed with the ability to estimate a patient's likelihood to be discharged given perioperative data.^73,74^ With incorporation of NLP and other complementary functions, these agents may be further able to perceive and address patient concerns. Other directions of AI applications further down the postoperative course have been proposed, whereby researchers commented on the potential of AI involvement in assisting with at-home patient care following discharge.^75,76^ Moving forwards, AI may grow into a key player in accompanying patients through their postoperative journey, simultaneously improving outcomes and alleviating utilization of finite healthcare resources.

### Educational Applications

AR, together with virtual reality (VR), also holds great benefits for medical trainees. Indeed, AR was first introduced as a teaching tool for penile implant placement among urology residents.^77^ By connecting real-world human gestures to the digital world, AR and VR are ideal channels for surgical education. Indeed, academic institutions such as the Mayo Clinic have already incorporated VR technologies into real-world surgical education.^78^ A review by Bilgic et al^79^ looked at the roles of AI in surgical education and highlighted a rising role of AI for the assessment and classification of surgical mastery, which may lead to the establishment of more objective competency metrics pertaining to breast reconstruction surgical techniques. Indeed, by being integrated into VR, AR, and/or robotic surgical simulators, AI may perform detailed analysis of kinematic information generated by the operator and return insightful feedback to improve surgical performance.^80^ This might of particular benefit to technically intricate areas such as microvascular breast reconstruction by expediting resident operative exposure and growth, especially with the recent advent of AR therewithin.^60^ Finally, plastic surgeons have demonstrated the potential of AI in contributing to scientific research,^81–84^ as well as performing on plastic surgery in-service examinations.^85,86^ Despite lingering ethical controversies, AI may evolve into a powerful scholarly adjunct to trainees in acquiring breast reconstruction knowledge and scientists in conducting breast reconstruction research, especially with active topics in need of further evidence (eg, BIA-ALCL, BIA-SCC, BII).^87–89^

## Discussion, Current Shortcomings, and Future Directions

Previous reviews of AI within breast surgery exist^5,90^; the present study actualizes and comprehensively synthesizes knowledge in this nascent field to provide breast reconstruction surgeons and researchers with a primer for subsequent endeavours. Although AI is a well-established scientific discipline, its integration within healthcare is a recent movement. As such, there is a dire need for further work, starting from proofs of concepts, to *in vivo* evaluations, to eventual clinical translation. As with any novel scientific field, time will be required to gather and synthesize evidence and progressively climb up the evidence pyramid. Moreover, in this field where new findings embody a high degree of inventiveness, the optimal medium of communicating novelty may need to be revisited. Patents may constitute appropriate and suitable complements to scientific publications, and their basic principles may be worth understanding.^91^ Indeed, premature publication strips the possibility for patent protection of potentially deserving intellectual property.^91^

Incorporation of AI technologies should be done earlier than later, as harsh learning curves will need to be tamed before the full potential and benefits of AI can be appreciated, as seen in robotic surgery.^92^ In so doing, awareness of the existence of assessment tools is crucial to ensure consistent progress.^93,94^ Once integral to the surgical workflow, AI has the potential to spur a veritable metamorphosis of current breast reconstruction care delivery and the healthcare system as a whole. By amping efficiency and bettering outcomes, greater numbers of patients in need could be seen, all the while maintaining high-quality standards. With breast reconstruction being a clear hotspot for health disparities,^95^ AI could constitute an elegant solution in flattening currently observed inequities.

That said, AI is not without its limitations, most of which are ethical and financial in nature. While the upfront cost of AI technologies has been brought up as a clear barrier,^49,96^ AI may ultimately reduce overall healthcare costs by optimizing both educational and clinical workflows.^97–99^ Moving forwards, the cost-effectiveness of AI—or lack thereof—will need to be ascertained through rigorous economic evaluations. As for ethics, access to sensitive health information by AI draws concerns related to patient privacy and confidentiality.^83^ Recent cases of malicious use of AI have been reported, where it was used to produce fake jurisprudence in legal defenses, as well as to generate child abuse images.^100–102^ In preventing against similar catastrophes of AI misuse in the healthcare setting, compliance to existing legislative frameworks such as the Health Insurance Portability and Accountability Act (HIPAA) in the United States, or General Data Protection Regulation (GDPR) in Europe, or the Personal Information Protection and Electronic Documents Act (PIPEDA) in Canada, and relevant regulations in other regions will be crucial to ensure responsible adoption of AI; de novo policymaking may also be warranted. With AI becoming increasingly authoritative with regard to clinical decision-making and surgical navigation, the incorporation of AI machinery as a proper member of the care team, and the resultant role of surgeons therein, will need to be revisited. Responsibility assumption in the occurrence of a medical error also becomes a subject of debate. Nevertheless, before all, it is prudent to suggest that the efficiency, efficacy, and reliability of AI be cemented with continued research and development to minimize the weight posed by current limitations. In so doing, collaborative efforts should aim to provide standardized and accessible AI training datasets,^66,90^ as well as validated and objective evaluation metrics and scales.^103^

## Conclusions

The territory yet unchartered by AI remains vast. Crystallized in this article is a review and discussion of current applications of AI in key domains of plastic and reconstructive breast surgery. With AI becoming ubiquitous in many aspects of human civilization, now, more than ever, is a crucial time for breast reconstruction surgeons to embrace this technology so as to not fall behind of an exponentially growing field. Aspiring trainees should seek to gain early familiarity and exposure, whereas established veterans should exercise adaptability and meet AI with their clinical experience to spark unique and valuable insights for optimal implementation.
